# A Pilot Study of the Combination of Entinostat with Capecitabine in Advanced Breast Cancer

**DOI:** 10.1155/2024/5515966

**Published:** 2024-02-07

**Authors:** Trish Millard, Christiana Brenin, Clare Humphrey, Ajay Dhakal, Carla Falkson, Gina Petroni, Nolan A. Wages, Patrick Dillon

**Affiliations:** ^1^Division of Hematology/Oncology, University of Virginia, Charlottesville, VA, USA; ^2^Division of Hematology/Oncology, University of Rochester Medical Center, Rochester, NY, USA; ^3^Department of Biostatistics, Virginia Commonwealth University, Richmond, VA, USA

## Abstract

**Background:**

Breast cancer has an unacceptably high recurrence rate when any residual disease is found following neoadjuvant treatment of high-risk disease. Based on clinical data suggesting an adjuvant role for epigenetic modifying agents in breast cancer and preclinical data suggesting synergistic activity of entinostat combined with capecitabine, we conducted a phase I, open-label study of these agents in metastatic breast cancer (MBC). Both agents have published doses for use in combination therapy, but the agents had not previously been combined with each other in a human trial.

**Methods:**

A multisite phase I dose escalation study was performed at two academic centers. Patients with pretreated, HER2-negative MBC, and measurable disease were enrolled. Dual dose escalation was performed via a Bayesian partial order continual assessment method. Dose levels ranged from entinostat 3 mg to 5 mg and capecitabine 800 mg/m^2^ to 1000 mg/m^2^.

**Results:**

Thirteen patients with MBC and a median of 4 lines of prior therapy were enrolled across four dose level combinations. The most common toxicities were neutropenia, thrombocytopenia, and palmar-plantar dysesthesia, which were expected toxicities. No new safety signals were observed. One dose-limiting toxicity was observed, which did not exceed a prespecified toxicity rate of 25%. The median treatment duration was 2.37 months. No partial nor complete responses were observed. The study was halted early prior to entering an expansion phase, due to drug supply limitations.

**Conclusion:**

The tested dosing combinations of entinostat and capecitabine are likely safe in heavily pretreated metastatic breast cancer. This study's clinical investigation of entinostat in breast cancer was halted, but drug development of this agent continues outside the US. There remains a need for postoperative adjuvant drug therapy for the subpopulation of breast cancer patients with high-risk residual cancer after curative therapy. This trial is registered with NCT03473639.

## 1. Introduction

Breast cancer is the most common cancer in women, and with improved screening methodology, more detection is occurring at earlier stages [1]. A subset of patients however undergo treatment with neoadjuvant chemotherapy prior to surgical resection, particularly those with locally advanced disease at presentation. The use of neoadjuvant therapy for the treatment of breast cancer is highly variable across breast cancer centers but has increased in recent years [2, 3]. Studies have shown that its use does not adversely impact disease-free or overall survival rates compared to chemotherapy given in the adjuvant setting, and thus, neoadjuvant chemotherapy is considered a safe and effective approach [4].

The disruption of epigenetic processes is an essential player in altering gene function and consequent malignant cellular transformation [5]. Histone deacetylases (HDAC) function in the posttranslational modification of DNA for regulation of gene expression and signal transduction and have been observed to be increased in cancer cells resulting in altered gene transcription, cell proliferation, and increased cell survival [6]. It is known that breast cancer, like most other forms of cancer, is epigenetically altered; thus, HDAC inhibitors have been developed to target such changes. HDAC inhibitors have been shown in multiple studies to result in the upregulation of thymidine phosphorylase with a synergistic effect when combined with fluoropyrimidines [7–11]. In vitro examination of multiple HDAC inhibitors, including entinostat, combined with capecitabine in breast cancer cell lines (MCF-7, MDA-MD-231, and MDA-MD-468) has shown time- and dose-dependent induction of thymidine phosphorylase and a synergistic antiproliferative effect [12, 13]. The mechanistic studies also found that thymidine synthase was downregulated by HDAC inhibitors which thereby blocks a major mechanism of capecitabine resistance in cancer cells [11, 13]. The findings of these studies provide the scientific basis for combining HDAC inhibitors with capecitabine in an effort to enhance efficacy and improve treatment outcomes. The combination of capecitabine and entinostat has not yet been studied in human clinical trials.

In the CREATE-X phase III clinical trial, patients (*n* = 910) with stage I-IIIB, HER2-negative breast cancer treated with neoadjuvant chemotherapy found at the time of surgery to have evidence of residual invasive disease or known node-positive disease were randomized to standard treatment with endocrine therapy as indicated or to treatment with 8 cycles of capecitabine (1250 mg/mg^2^ bid D1-14, q21 days). The concomitant administration of postsurgical endocrine therapy in the capecitabine group was allowed. The tolerability was consistent with the established safety profile of capecitabine, and relative dose intensity was reported as 78.7% for patients receiving the full eight cycles of therapy. At three years, there was a statistically significant improvement in disease-free survival (82.8% vs. 73.9%, *p* = 0.01) and overall survival (94.0% vs. 88.9%, *p* = 0.01) in patients treated with capecitabine [14].

Entinostat, an oral class 1 isoform selective HDAC I and IV inhibitor, was granted FDA breakthrough status for advanced breast cancer in 2013 [15]. The mechanism of action for entinostat is that it upregulates histone acetylation. Furthermore, entinostat appears to serve as an agent to revert chromatin winding/structure in ways that allow for more normal expression of key tumor suppressor genes which likely were turned off by the carcinogenesis process. Targets of entinostat have been published and include other some proapoptotic genes and signaling pathway genes. It is thought that the summation of effects is a return of the ability of the cell to control normal cell-cycle arrest and therefore revert to a less malignant phenotype. Entinostat showed positive phase II data in metastatic breast cancer in the ENCORE301 study [16]. Entinostat has since been studied in two randomized phase III clinical trials in combination with exemestane in metastatic breast cancer. In the cooperative group E2112 study of 706 patients, there was not a statistically significant improvement in PFS or OS compared to exemestane alone [17]. In a concomitant randomized phase III study in 354 Chinese women with MBC, there was a 2.6 month PFS benefit (*p* = 0.046) favoring entinostat plus exemestane [18]. Reasons for the discrepant results from nearly identical phase III trials have been reviewed elsewhere [18].

In this study, we assessed the safety and tolerability of the combination of entinostat and capecitabine at various dose combinations. As discussed below in the methods, the study was secondarily designed to assess the disease-free survival (DFS) after use of entinostat and capecitabine in high-risk breast cancer patients with residual disease after completion of neoadjuvant chemotherapy and definitive surgery. The key hypothesis for the second part of the study was that the addition of entinostat to standard adjuvant capecitabine would improve DFS in this population.

## 2. Methods

### 2.1. Patient Population

The study had two phases, a safety dose finding lead-in and second part efficacy study in the population of breast cancer patients with residual disease after neoadjuvant chemotherapy. The dose finding lead-in was required by regulatory authorities due to the absence of published safety data on the combination of capecitabine and entinostat. Details regarding the modeling approach and design considerations for both phases of the study have been provided in a prior report [19]. Due to drug supply limitations, the study closed after the first phase. For the first phase safety lead-in, metastatic breast cancer patients were enrolled. The patient population included female patients age 18 years and older, ECOG performance status of 0-2, with histologically confirmed diagnosis of metastatic, ER/PR-positive or negative, and HER2-negative breast cancer.

### 2.2. Study Objectives

The primary objective of this study was the measurement of toxicity and safety as defined by the incidence and severity of adverse events and the number of patients discontinuing treatment due to adverse events. Secondary objectives consisted of measuring invasive breast cancer recurrence, disease-free survival, and overall survival. A correlative outcome was also planned by measurement of circulating tumor DNA (ctDNA) and circulating tumor cells (CTCs) as a means of assessing disease activity before and after adjuvant chemotherapy.

### 2.3. Study Design, Dose Allocation, and Statistical Considerations

The first phase of this trial was designed to determine the maximum tolerated dose combination (MTDC), defined by an acceptable toxicity profile of the combination, from among the dose combinations in Table 1.

This study was implemented at two large academic medical centers, the University of Virginia Health System and University of Rochester Medical Center. The study was an open-label pilot study. For the first lead-in phase, patients with metastatic disease were treated with capecitabine and entinostat at varied dosing combinations. Notably the existing dose for entinostat monotherapy at the time of study conception was 5 mg po weekly. The starting dose for entinostat used in this combination study was 3 mg po weekly based on preclinical and phase 1 data [20, 21]. The starting dose for capecitabine for combination in this study was 800 mg/m^2^ twice daily for 14 days followed by 7 days off (which falls on the low end of the range of clinically utilized and validated doses for capecitabine in combination). This starting dose was chosen as a safe dose with reasonable expectation of potential for clinical activity. The maximum target doses were entinostat 5 mg po weekly and capecitabine 1000 mg/m^2^ po bid 14 days on, 7 days off. The maximum target dose for capecitabine was selected based on prevailing clinical practice at the time of study design and in anticipation of possible overlap of toxicities.

Dose escalation was conducted using the Bayesian partial order continual reassessment method (POCRM) for drug combinations [22]. Dose-limiting toxicity (DLT) was determined by adverse events occurring during the first cycle of treatment. The MTDC per protocol was specified to be the drug dose combination with a rate of DLT nearest to the target rate of 25%. Patients were monitored for toxicity using the standard NCI CTCAE version 4.03 tool. Individual patients experiencing DLT were required to interrupt therapy and reduce dose. Each occurrence of a DLT was then used to recalculate probabilities of further toxicity at all dose levels and to guide assignment of subsequent patients to treatment levels. The statistical model was set to allocate subsequent participants to the dose combination with an estimated future DLT rate closest to 25%.

Per published designs for a two-drug combinatorial study in cancer, a 90% confidence interval would be calculated around the DLT probability for each two-drug combination level studied. The final recommended dose combination to move forward to the second phase would then be the best dose level with a DLT rate under 25%. Estimates were made using the continual reassessment method (CRM) models [23]. The second phase of the study was designed to continue until 30 eligible participants with high-risk residual disease had been treated with protocol treatment at the recommended MTDC. The maximum target sample size was based upon acquiring sufficient information to assess the goal of determining the MTDC in participants with high-risk residual disease, obtaining an estimate of treatment tolerance and preliminary assessment of disease-free survival. The enrollment goal of 30 breast cancer subjects was calculated/powered to test for tolerance. The null rate of treatment tolerability was 60% and was to be compared to an alternative rate of 80% with a one-sided type I error rate of 0.094 and power of 0.871 with a binomial test. The choices of the null and alternative rates were based upon results reported in the CREATE-X trial [14] which reported 75% of participants (95% CI [69, 80%]) treated with 8 cycles of capecitabine did not discontinue treatment. For this study, data supporting a tolerance rate of 60% (below the lower limit of the confidence bound) would be considered unacceptable. At study conclusion, frequency, proportion, and severity of adverse events and DLTs by treatment combination were tabulated.

The study was an investigator-initiated trial funded by the UVA Cancer Center and philanthropic funds. The trial was overseen by the institutional review board, the protocol review board, and the data safety board at the UVA Cancer Center. The study was compliant with ICH-GCP guidelines. All consented patients have been included in this report.

## 3. Results

To assess safety and tolerability, thirteen patients with pretreated metastatic breast cancer (median of 4 prior therapies) who met full inclusion criteria were assigned by the POCRM [22] to one of the four treatment arms with differing dose combinations of entinostat and capecitabine. The median treatment duration was 2.37 months. The median follow-up time was 2.96 months. Evaluation for true progression-free survival and overall survival was not reported due to patients lost to long-term follow-up. Notably no differences in treatment duration were observed by receptor subtype nor by arm of study, although the study was not powered to measure such differences. In this study, the primary hormone receptor status of the participants was 69% ER positive and 31% triple negative (Table 2). All patients were metastatic (Table 3), although at original premetastatic diagnosis, 15% of participants were node negative, 38% were N1, 15% were N2, 7% were N3, and 23% were missing. None of the triple negative patients had received prior immunotherapy.

Subjects in all four of the dose combination arms were monitored for adverse events based upon a Bayesian POCRM for drug combinations, with arms exceeding the target rate of 25% deemed too toxic of a combination [16]. In arm C1, there were three patients total, with no DLTs observed. Arm C2 consisted of four patients, with one DLT observed, equaling the target rate of 25%. Arm C3 consisted of four patients, with no DLTs observed. Arm C4 had two patients, with no DLTs observed. Groups C1, C2, and C4 had DLTs less than 25% of the time. None of the four arms had an observed DLT rate above the target rate of 25% (Table 4).

The most common reported adverse events (Table 5) were decreased platelet count and decreased white cell count in 61.5% and 53.8% of participants, respectively. Surprisingly, palmar-plantar erythrodysesthesia (hand-foot syndrome) was only seen in 3 patients, thus suggesting that total exposure time and dosage for capecitabine were low in this heavily pretreated population. Low grade diarrhea was observed at the expected rate of around 50%.

## 4. Discussion

Entinostat was granted FDA breakthrough status for advanced breast cancer in 2013 based on positive efficacy results from the phase II study ENCORE 301 combining entinostat with an aromatase inhibitor (AI), as well as encouraging results from other phase II studies [16, 24–27]. Entinostat was further tested in the E2112 phase III study and the accompanying Chinese phase III study, both of which compared AI plus entinostat to AI alone in metastatic disease [17, 18, 28]. The study results are conflicting, but studies of entinostat are continuing outside the US. Likewise, studies of effects of entinostat on immunotherapy of breast cancer are ongoing [29]. Entinostat has been administered to nearly 1500 patients in early and late phase cancer studies, and the drug appears well tolerated.

This trial was designed to examine the safety and tolerability of the combination of entinostat with capecitabine by utilizing a Bayesian POCRM for drug combinations [22]. This design follows a novel approach to early-phase oncology treatment trials. A toxicity was considered a DLT if it occurred during the first cycle of treatment (inclusive of labs drawn on day #22) and is considered to be probably or definitely related to treatment. The occurrence of DLTs drove the escalation and stopping decisions. In this study, all four of the arms tested with combinations of entinostat and capecitabine were found to have a DLT occurrence ≤ 25%, indicating sufficient tolerability and safety in all groups. These findings suggest the likely safe combination of these agents in metastatic breast cancer patients. This is particularly notable given that the patients in the present study were heavily pretreated and therefore more prone to tolerance issues such as myelosuppression.

The most common adverse events for patients receiving entinostat monotherapy, regardless of tumor type, are fatigue, gastrointestinal disturbance, thrombocytopenia, anemia, and hypoalbuminemia. In the present study, the adverse events are typical of those seen with entinostat and capecitabine monotherapy, suggesting that the combination doses did not lead to excess compounded toxicity. Arm C2 (entinostat 5 mg, capecitabine 800 mg/m^2^) was the only arm in which a DLT was observed (1/4 DLTs; 25%). The study design would have allowed for the accrual of more patients to verify the toxicity signal, but the study stopped before this particular cohort was expanded far enough to make definitive conclusions about this combination level. A criticism of the study could be that other lower and higher dose combinations of these drugs were not tested. Upon review of the dose levels chosen, outside experts deemed the chosen doses to be pragmatic and safe combinations to explore. Entinostat doses lower than 3 mg were previously not active, and capecitabine doses lower than 800 mg/m^2^ were felt to be subtherapeutic.

Limitations of this study include the fact that it stopped earlier than designed due to lack of drug availability. The study was also defined by study in a heavily pretreated patient population. Thus, it is difficult to assess whether similar toxicity would have occurred in less heavily treated patients. Finally, the study is impacted by shifts in treatment paradigms in metastatic breast cancer. With the development of CDK4/6 inhibitors, PI3Kinase inhibitors, checkpoint inhibitors, and antibody-drug conjugates, there are many developing treatment options available to metastatic breast cancer patients, thus rendering application of these results unclear in the current breast cancer treatment landscape.

This study was designed to ultimately study the unique high-risk patients with residual disease after neoadjuvant therapy who are known to have an unmet treatment need. Unfortunately, this phase of the study was not able to be completed. Future studies in this cohort of patients should be considered. Given that entinostat plus capecitabine was tolerated in a variety of dosing combinations in heavily pretreated patients with metastatic disease, it is reasonable to predict that such a combination could also be tolerated in patients with stage I-III breast cancer with residual disease after neoadjuvant therapy ± radiation therapy. Furthermore, this study presents an early signal of safety of the combination of entinostat and capecitabine, which may serve as a basis for study in other malignancies with unmet treatment needs.

## 5. Conclusion

The use of neoadjuvant therapy for breast cancer treatment is highly variable across breast cancer centers but has notably increased in recent years. There is now an approved standard therapy for a subset of patients with stage I-III HER2-negative and ER/PR-positive breast cancer with residual disease (abemaciclib as per FDA label and Monarch-E result). Given the high-risk nature of this patient population and the multitude of targeted agents now approved in the metastatic setting, additional trials in the residual disease setting are warranted, to include novel agents and circulating tumor DNA studies. Our study demonstrated that combinations of entinostat and capecitabine in advanced metastatic breast cancer are safe and tolerable over the range of dose combinations studied.

## Figures and Tables

**Table 1:** Dose combinations studied in the pilot trial.

Combination designation			
Entinostat	5 mg	Combination 2	Combination 4
	3 mg	Combination 1	Combination 3
		800 mg/m^2^	1000 mg/m^2^
Capecitabine	

**Table 2:** Baseline characteristics of study population.

Baseline characteristics	Arm			
	C1	C2	C3	C4
Age	67.7	66.3	62.7	70.0
Black/African American	1	0	0	1
Caucasian	2	4	4	1
ECOG PS				
0—fully active	1	3	1	1
1—restricted	1	1	3	1
Receptor status				
ER and PR positive	2	3	2	2
Triple negative	1	1	2	0
Number per arm	3	4	4	2

**Table 3:** Summary of sites of metastases in study participants. Note that totals do not equal to 13 due to presence of multiple metastases in participants.

Metastasis summary	Total	Arm			
		C1	C2	C3	C4
	*N*	*N*	*N*	*N*	*N*
Bone	8	1	2	4	1
Liver	6	1	2	1	2
Lung	1	0	0	1	0
Lymph nodes	3	1	1	0	1
Other	4	1	0	3	0

**Table 4:** Summary dose-limiting toxicity table.

Arm	Arm description	# pts w/ DLT	# pts total	%	CI lower	CI upper
C1	Entinostat 3 mg, capecitabine 800 mg	0	3	0	0	63.2
C2	Entinostat 5 mg, capecitabine 800 mg	1	4	25	1.3	75.1
C3	Entinostat 3 mg, capecitabine 1000 mg	0	4	0	0	52.7
C4	Entinostat 5 mg, capecitabine 1000 mg	0	2	0	0	77.6

**Table 5:** Summary of adverse events by grade per CTCAE v. 4.03.

AE	G1	G2	G3	G4	Total
Anemia	5	1			6
Abdominal pain	1				1
Diarrhea	3	4			7
Dyspepsia	2				2
Mucositis oral			1		1
Nausea	3	1			4
Fatigue	2	2	1		5
White blood cell decreased	1	6			7
Anorexia	4				4
Peripheral sensory neuropathy		1			1
Dyspnea	2	1			3
Palmar-plantar erythrodysesthesia	2	1			3
Hypertension		1			1
Thromboembolic event		1			1
Thrombocytopenia	7	1			8

## Data Availability

The deidentified radiology, laboratory, toxicity, and pathology data used to support the findings of this study may be released upon application to the University of Virginia Institutional review board for Health Science Research, who can be contacted at irbhsr@virginia.edu or 1-434-924-2620.
